# Supplementary material for: Long lasting anti-IgG chikungunya seropositivity in the Mayotte population will not be enough to prevent future outbreaks: A seroprevalence study, 2019
Source: PLoS One. 2023 May 18;18(5):e0285879. doi: 10.1371/journal.pone.0285879 (PMC10194921; doi:10.1371/journal.pone.0285879)
Supplement: S1 File — (DOCX) [file pone.0285879.s001.docx]

**Supplementary information**

S 1 Map of Mayotte with communes and corresponding sectors in the table on the right

| **Sectors** | **Communes** |
| --- | --- |
| Centre | Chiconi |
| Centre | Dembeni |
| Centre | Ouangani |
| Centre | Sada |
| Centre | Tsingoni |
|  |  |
| Mamoudzou | Mamoudzou |
|  |  |
| North | Acqua |
| North | Bandraboua |
| North | Koungou |
| North | Mtsamboro |
| North | Mtsangamouji |
|  |  |
| South | Bandrele |
| South | Boueni |
| South | Chirongui |
| South | Kani-Keli |
|  |  |
| Petite-Terre | Dzaoudzi -Labattoir |
| Petite-Terre | Pamandzi |


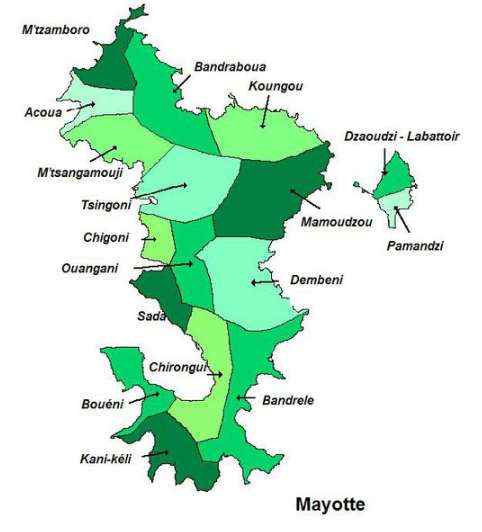


S 2
