# Supplementary material for: Long lasting anti-IgG chikungunya seropositivity in the Mayotte population will not be enough to prevent future outbreaks: A seroprevalence study, 2019
Source: PLoS One. 2023 May 18;18(5):e0285879. doi: 10.1371/journal.pone.0285879 (PMC10194921; doi:10.1371/journal.pone.0285879)
Supplement: S2 File — (DOCX) [file pone.0285879.s002.docx]

**Supplementary information**

**S2**. Weighed chikungunya seroprevalence, weighed prevalence ratios and univariate analysis for all covariates (*N=* ***1438,*** *it may slightly differ for some of the covariates due to missing values).*

|  |  | |  | |  |  |  |  |  |  |  |  | **Univariate**  **(Poisson regression)** | | | |
| --- | --- | --- | --- | --- | --- | --- | --- | --- | --- | --- | --- | --- | --- | --- | --- | --- |
|  |  | **Tot** | | **%** | | **N CHIK  negative** | **N CHIK  positive** | **N CHIK  positive (%)** | **CHIK + weighed prevalence (%)** | **95 % CI** | | **P value** | **wPR** | **95 % CI** | | **P value** |
| **Sociodemographic factors** | | | | | |  |  |  |  |  |  |  |  |  |  |  |
| Age (Years) | 15-17 | 126 | | 8.76 | | 83 | 43 | 34.13 | 35.9 | 26.44 | 46.61 | 0.787 |  |  |  |  |
|  | 18-29 | 370 | | 25.73 | | 223 | 147 | 39.73 | 36.97 | 31.08 | 43.27 |  |  |  |  |  |
|  | 30-49 | 648 | | 45.06 | | 417 | 231 | 35.65 | 34.1 | 29.55 | 38.97 |  |  |  |  |  |
|  | 50-69 | 294 | | 20.45 | | 209 | 85 | 28.91 | 32.09 | 25.28 | 39.76 |  |  |  |  |  |
| Sex | Male | 455 | | 31.64 | | 290 | 165 | 36.26 | 35.45 | 30.40 | 40.85 | 0.767 |  |  |  |  |
|  | Female | 983 | | 68.36 | | 642 | 341 | 34.69 | 65.54 | 30.63 | 38.17 |  |  |  |  |  |
| Sector of residence | CENTRE | 401 | | 27.89 | | 274 | 127 | **31.67** | 30.64 | 25.38 | 36.46 | 0.000 | Ref |  |  |  |
|  | MAMOUDZOU | 518 | | 36.02 | | 288 | 230 | 44.40 | 43.84 | 38.45 | 49.38 |  | 1.43 | 1.15 | 1.78 | 0.001 |
|  | NORD | 143 | | 9.94 | | 84 | 59 | 41.26 | 43.09 | 34.15 | 52.52 |  | 1.41 | 1.06 | 1.86 | 0.018 |
|  | PETITE-TERRE | 218 | | 15.16 | | 157 | 61 | 27.98 | 30.42 | 23.40 | 38.49 |  | 0.99 | 0.73 | 1.35 | 0.964 |
|  | SUD | 158 | | 10.99 | | 129 | 29 | 18.35 | 14.26 | 9.35 | 21.16 |  | 0.47 | 0.30 | 0.73 | 0.001 |
| No of children | 1-3 | 480 | | 33.38 | | 312 | 168 | 35.00 | 30.54 | 25.53 | 36.03 | 0.176 | ref |  |  |  |
|  | 4 or more | 589 | | 40.96 | | 385 | 204 | 34.63 | 35.97 | 31.07 | 41.18 |  | 1.18 | 0.94 | 1.47 | 0.151 |
|  | UNK | 369 | | 25.66 | | 235 | 134 | 36.31 | 37.88 | 31.91 | 44.25 |  | 1.24 | 0.98 | 1.57 | 0.076 |
| Education level | No education | 913 | | 63.58 | | 557 | 356 | 38.99 | 39.04 | 34.96 | 43.28 | 0.000 | ref |  |  |  |
|  | Education below university degree | 387 | | 26.95 | | 267 | 120 | 31.01 | 34.06 | 28.28 | 40.37 |  | 0.87 | 0.71 | 1.07 | 0.198 |
|  | University degree/ higher education | 103 | | 7.17 | | 84 | 19 | 18.45 | 13.91 | 8.06 | 22.95 |  | 0.36 | 0.21 | 0.61 | 0.000 |
|  | Other education | 33 | | 2.30 | | 23 | 10 | 30.30 | 25.37 | 12.10 | 45.65 |  | 0.65 | 0.33 | 1.29 | 0.216 |
| Professional situation | Employed | 328 | | 22.86 | | 241 | 87 | 26.52 | 23.88 | 18.71 | 29.96 | 0.005 | ref |  |  |  |
|  | Unemployed | 175 | | 12.20 | | 112 | 63 | 36.00 | 37.96 | 29.68 | 47.01 |  | 1.59 | 1.14 | 2.21 | 0.006 |
|  | Student | 270 | | 18.82 | | 156 | 114 | 42.22 | 39.78 | 32.46 | 47.58 |  | 1.67 | 1.22 | 2.26 | 0.001 |
|  | Inactive (pensioner, housekeeping person etc,) | 627 | | 43.69 | | 401 | 226 | 36.04 | 37.19 | 32.28 | 42.38 |  | 1.56 | 1.19 | 2.05 | 0.001 |
|  | Paid stage or other | 35 | | 2.44 | | 20 | 15 | 42.86 | 46.82 | 28.67 | 65.86 |  | 1.96 | 1.21 | 3.17 | 0.006 |
| Social security | No social security | 555 | | 38.60 | | 324 | 231 | 41.62 | 40.26 | 35.18 | 45.56 | 0.002 |  |  |  |  |
|  | Only social security | 778 | | 54.10 | | 523 | 255 | 32.78 | 33.61 | 29.38 | 38.11 |  | 0.83 | 0.69 | 1.00 | 0.054 |
|  | Social security and complementary assurance | 105 | | 7.30 | | 85 | 20 | 19.05 | 17.43 | 9.97 | 28.7 |  | 0.43 | 0.25 | 0.75 | 0.003 |
| Place of birth | Mayotte | 552 | | 38.41 | | 401 | 151 | 27.36 | 27.85 | 23.35 | 32.84 | 0.000 | ref |  |  |  |
|  | Les Comores | 754 | | 52.47 | | 430 | 324 | 42.97 | 43.42 | 38.86 | 48.09 |  | 1.56 | 1.28 | 1.91 | 0.000 |
|  | Madagascar | 74 | | 5.15 | | 51 | 23 | 31.08 | 28.49 | 15.92 | 45.59 |  | 1.02 | 0.59 | 1.79 | 0.937 |
|  | Metropolitan France and other countries | 57 | | 3.97 | | 49 | 8 | 14.04 | 11.67 | 4.63 | 26.41 |  | 0.42 | 0.17 | 1.03 | 0.058 |
| Time spent in Mayotte | Up to 9 years in Mayotte | 334 | | 23.23 | | 209 | 125 | 37.43 | 35.26 | 28.94 | 42.14 | 0.000 | ref |  |  |  |
|  | 10 years and more | 549 | | 38.18 | | 320 | 229 | 41.71 | 43.48 | 38.10 | 49.02 |  | 1.23 | 0.98 | 1.55 | 0.069 |
|  | UNK | 555 | | 38.60 | | 403 | 152 | 27.39 | 27.77 | 23.29 | 32.74 |  | 0.79 | 0.61 | 1.02 | 0.065 |
| Number of household members | 1 to 3 | 623 | | 43.32 | | 405 | 218 | 34.99 | 35.57 | 30.77 | 40.68 | 0.110 | ref |  |  |  |
|  | 4 to 5 | 439 | | 30.53 | | 293 | 146 | 33.26 | 30.32 | 25.17 | 36.02 |  | 0.85 | 0.68 | 1.07 | 0.169 |
|  | 6 to 13 | 376 | | 26.15 | | 234 | 142 | 37.77 | 38.41 | 32.69 | 44.48 |  | 1.08 | 0.88 | 1.33 | 0.469 |
| **Household & environment** | | | | | |  |  |  |  |  |  |  |  |  |  |  |
| Habitation type | House on cement | 742 | | 51.60 | | 537 | 205 | 27.63 | 26.09 | 22.22 | 30.37 | 0.000 | ref |  |  |  |
|  | Traditional wooden house or wood-sheet metal hut | 581 | | 40.40 | | 325 | 256 | 44.06 | 45.45 | 40.36 | 50.64 |  | 1.74 | 1.43 | 2.11 | 0.000 |
|  | Apartment | 115 | | 8.00 | | 70 | 45 | 39.13 | 34.19 | 23.64 | 46.57 |  | 1.31 | 0.90 | 1.91 | 0.157 |
| Running water in the household | No | 712 | | 49.51 | | 408 | 304 | 42.70 | 44.76 | 40.09 | 49.53 | 0.000 | ref |  |  |  |
|  | Yes | 726 | | 50.49 | | 524 | 202 | 27.82 | 25.68 | 21.84 | 29.95 |  | 0.57 | 0.47 | 0.69 | 0.000 |
| WC in the household | No | 744 | | 51.74 | | 429 | 315 | 42.34 | 44.58 | 40.04 | 49.21 | 0.000 | ref |  |  |  |
|  | Yes | 694 | | 48.26 | | 503 | 191 | 27.52 | 24.37 | 20.55 | 28.63 |  | 0.57 | 0.47 | 0.69 | 0.000 |
| Running water and WC in the household | (interaction term) running water and WC in household |  | |  | |  |  |  |  |  |  |  |  |  |  |  |
| Go to the river for bathing | Never | 1336 | | 92.97 | | 879 | 457 | 34.21 | 33.92 | 30.69 | 37.3 | 0.024 | ref |  |  |  |
|  | Sometimes/often | 61 | | 4.24 | | 32 | 29 | 47.54 | 44.12 | 29.69 | 59.62 |  | 1.3 | 0.91 | 1.87 | 0.155 |
|  | Always | 40 | | 2.78 | | 20 | 20 | 50.00 | 55.64 | 37.11 | 72.73 |  | 1.64 | 1.15 | 2.32 | 0.005 |
| Go to the river for washing clothes or dishes | Never | 1247 | | 86.72 | | 824 | 423 | 33.92 | 33.46 | 30.13 | 36.96 | 0.055 | ref |  |  |  |
|  | Sometimes/often for either reason | 160 | | 11.13 | | 92 | 68 | 42.50 | 44.24 | 34.75 | 54.17 |  | 1.32 | 1.04 | 1.69 | 0.025 |
|  | Always | 31 | | 2.16 | | 16 | 15 | 48.39 | 47 | 27.37 | 67.61 |  | 1.4 | 0.88 | 2.22 | 0.152 |
| Waste: Presence of an external personal closed bin | No | 1058 | | 73.63 | | 675 | 383 | 36.20 | 36.07 | 32.42 | 39.88 | 0.245 |  |  |  |  |
|  | Yes | 379 | | 26.37 | | 256 | 123 | 32.45 | 31.7 | 25.85 | 38.2 |  |  |  |  |  |
| Waste: Presence of an external collective bin | No | 1057 | | 73.61 | | 675 | 382 | 36.14 | 36.05 | 32.40 | 39.86 | 0.247 |  |  |  |  |
|  | Yes | 379 | | 26.39 | | 256 | 123 | 32.45 | 31.7 | 31.77 | 38.17 |  |  |  |  |  |
| **Attitudes to mosquitoes born disease prevention** | | | | | |  |  |  |  |  |  |  |  |  |  |  |
| Perception about mosquitoes | Not a problem | 171 | | 11.95 | | 121 | 50 | 29.24 | 25.62 | 17.93 | 35.21 | 0.017 | ref |  |  |  |
|  | A bit annoyed | 173 | | 12.09 | | 116 | 57 | 32.95 | 27.97 | 20.58 | 36.8 |  | 1.09 | 0.70 | 1.71 | 0.700 |
|  | Annoyed | 160 | | 11.18 | | 110 | 50 | 31.25 | 30.59 | 22.17 | 40.54 |  | 1.19 | 0.76 | 1.89 | 0.445 |
|  | Very annoyed | 927 | | 64.78 | | 581 | 346 | 37.32 | 34.88 | 34.57 | 42.69 |  | 1.5 | 1.05 | 2.14 | 0.024 |
| Understanding whether transmission of malaria is via mosquitoes | No | 170 | | 11.82 | | 120 | 50 | 29.41 | 24.7 | 17.67 | 33.4 | 0.060 | ref |  |  |  |
|  | Yes | 960 | | 66.76 | | 606 | 354 | 36.88 | 36.74 | 32.88 | 40.77 |  | 1.49 | 1.06 | 2.08 | 0.021 |
|  | no answer | 308 | | 21.42 | | 206 | 102 | 33.12 | 34.91 | 27.96 | 42.04 |  | 1.4 | 0.96 | 2.05 | 0.080 |
| Concerns about getting a mosquito-borne diseases | No | 180 | | 12.52 | | 121 | 59 | 32.78 | 32.6 | 24.53 | 41.87 | 0.744 |  |  |  |  |
|  | Yes | 1209 | | 84.08 | | 777 | 432 | 35.73 | 35.08 | 31.68 | 38.64 |  |  |  |  |  |
|  | No answer | 49 | | 3.41 | | 34 | 15 | 30.61 | 40.8 | 22.36 | 62.26 |  |  |  |  |  |
| Perception about malaria | No serious or not so serious | 45 | | 3.13 | | 31 | 14 | 31.11 | 30.75 | 16.70 | 49.58 | 0.584 |  |  |  |  |
|  | Very serious disease | 1258 | | 87.48 | | 821 | 437 | 34.74 | 34.53 | 31.21 | 38.02 |  |  |  |  |  |
|  | I don't know | 135 | | 9.39 | | 80 | 55 | 40.74 | 39.75 | 29.35 | 51.18 |  |  |  |  |  |
| **Protective measures** | | | | | |  |  |  |  |  |  |  |  |  |  |  |
| Personal repellents for skin or for clothes | No | 1110 | | 77.19 | | 702 | 408 | 36.76 | 36.13 | 32.55 | 39.86 | 0.190 | ref |  |  |  |
|  | Yes | 328 | | 22.81 | | 230 | 98 | 29.88 | 30.94 | 24.71 | 37.95 |  | 0.86 | 0.68 | 1.09 | 0.201 |
| Use of insecticides (serpentines and alike) | No | 1033 | | 71.84 | | 669 | 364 | 35.24 | 34.54 | 30.91 | 38.35 | 0.714 |  |  |  |  |
|  | Yes | 405 | | 28.16 | | 263 | 142 | 35.06 | 35.89 | 29.92 | 42.33 |  |  |  |  |  |
| Covering one-self with appropriate clothes | No | 1411 | | 98.12 | | 919 | 492 | 34.87 | 34.8 | 31.63 | 38.1 | 0.609 |  |  |  |  |
|  | Yes | 27 | | 1.88 | | 13 | 14 | 51.85 | 40.32 | 21.64 | 62.3 |  |  |  |  |  |
| Nets for beds and window screenings | No | 634 | | 44.09 | | 409 | 225 | 35.49 | 35.24 | 30.53 | 40.25 | 0.862 |  |  |  |  |
|  | Yes | 804 | | 55.91 | | 523 | 281 | 34.95 | 34.66 | 30.54 | 39.06 |  |  |  |  |  |
| Air conditioning | No | 662 | | 46.04 | | 421 | 241 | 36.40 | 37.39 | 32.74 | 42.28 | 0.162 | ref |  |  |  |
|  | Yes | 776 | | 53.96 | | 511 | 265 | 34.15 | 32.8 | 28.67 | 37.23 |  | 0.88 | 0.73 | 1.05 | 0.161 |
| No protection at all | No, I do protect myself | 1317 | | 91.59 | | 850 | 467 | 35.46 | 34.99 | 31.72 | 38.41 | 0.845 |  |  |  |  |
|  | Yes, no protection | 121 | | 8.41 | | 82 | 39 | 32.23 | 33.84 | 23.77 | 38.17 |  |  |  |  |  |
| **Vector control** | | | | | |  |  |  |  |  |  |  |  |  |  |  |
| Being concerned & Elimination of stagnant water | Not concerned and not eliminating stagnant water | 273 | | 18.98 | | 171 | 102 | 37.36 | 37.53 | 30.50 | 45.14 | 0.061 | ref |  |  |  |
|  | I am not concerned about vector control but I eliminate stagnant water | 727 | | 50.56 | | 461 | 266 | 36.59 | 37.26 | 32.84 | 41.89 |  | 0.99 | 0.77 | 1.25 | 0.95 |
|  | I am concerned but I don't eliminate stagnant water | 438 | | 30.46 | | 300 | 138 | 31.51 | 28.88 | 23.64 | 34.74 |  | 0.77 | 0.58 | 1.01 | 0.062 |
| Covering of water reservoirs | No | 1109 | | 77.12 | | 728 | 381 | 34.36 | 32.88 | 29.39 | 39.56 | 0.021 | ref |  |  |  |
|  | Yes | 329 | | 22.88 | | 204 | 125 | 37.99 | 41.77 | 35.11 | 48.75 |  | 1.27 | 1.04 | 1.55 | 0.017 |
| Treatment of water reservoirs | No | 1395 | | 97.01 | | 900 | 495 | 35.48 | 35.19 | 32.00 | 38.52 | 0.366 |  |  |  |  |
|  | Yes | 43 | | 2.99 | | 32 | 11 | 25.58 | 27.26 | 14.46 | 45.38 |  |  |  |  |  |
| Not applicable in my house | No, it would be applicable | 1277 | | 88.80 | | 828 | 449 | 35.16 | 34.97 | 31.64 | 38.45 | 0.913 |  |  |  |  |
|  | Yes, nothing can be done | 161 | | 11.20 | | 104 | 57 | 35.40 | 34.41 | 25.73 | 44.27 |  |  |  |  |  |
| Other measures | No | 1425 | | 99.10 | | 924 | 501 | 35.16 | 35 | 31.85 | 38.28 | 0.387 |  |  |  |  |
|  | Yes | 13 | | 0.90 | | 8 | 5 | 38.46 | 22.18 | 6.20 | 55.11 |  |  |  |  |  |
| **Health** |  |  | |  | |  |  |  |  |  |  |  |  |  |  |  |
| Perception of personal health | Excellent, very good | 190 | | 13.23 | | 124 | 66 | 34.74 | 34.08 | 26.04 | 43.16 | 0.479 |  |  |  |  |
|  | Reasonably healthy and good health status | 936 | | 65.18 | | 619 | 317 | 33.87 | 33.58 | 29.79 | 37.61 |  |  |  |  |  |
|  | Bad, very bad health | 273 | | 19.01 | | 163 | 110 | 40.29 | 40.4 | 32.91 | 48.38 |  |  |  |  |  |
|  | No answer or I can't say | 37 | | 2.58 | | 24 | 13 | 35.14 | 38.53 | 21.74 | 58.59 |  |  |  |  |  |
| Limitations due to health conditions | No limitations | 1169 | | 81.81 | | 770 | 399 | 34.13 | 33.49 | 30.10 | 37.07 | 0.179 | ref |  |  |  |
|  | Yes, very limited | 78 | | 5.46 | | 46 | 32 | 41.03 | 42.31 | 28.86 | 57.01 |  | 1.26 | 0.88 | 1.81 | 0.200 |
|  | Limited to some extent | 182 | | 12.74 | | 111 | 71 | 39.01 | 41.44 | 31.76 | 51.2 |  | 1.22 | 0.95 | 1.59 | 0.123 |
| Presence of chronic diseases | No | 945 | | 65.81 | | 602 | 343 | 36.30 | 35.45 | 31.65 | 39.45 | 0.362 |  |  |  |  |
|  | Yes | 456 | | 31.75 | | 301 | 155 | 33.99 | 34.9 | 29.28 | 40.98 |  |  |  |  |  |
|  | I can't say | 35 | | 2.44 | | 27 | 8 | 22.86 | 20.74 | 31.82 | 38.23 |  |  |  |  |  |
| Reported as diagnosed with diabetes | No | 1308 | | 90.96 | | 845 | 463 | 35.40 | 35.36 | 32.07 | 38.8 | 0.403 |  |  |  |  |
|  | yes | 107 | | 7.44 | | 74 | 33 | 30.84 | 27.56 | 18.02 | 39.7 |  |  |  |  |  |
|  | I don't know | 23 | | 1.60 | | 13 | 10 | 43.48 | 39.41 | 31.77 | 38.17 |  |  |  |  |  |
| Reported as diagnosed with hypertension | No | 1104 | | 76.77 | | 704 | 400 | 36.23 | 35.18 | 31.61 | 38.92 | 0.983 |  |  |  |  |
|  | yes | 311 | | 21.63 | | 212 | 99 | 31.83 | 33.94 | 27.53 | 40.99 |  |  |  |  |  |
|  | I don't know | 23 | | 1.60 | | 16 | 7 | 30.43 | 31.6 | 14.55 | 55.63 |  |  |  |  |  |
|  | Never drunk | 1169 | | 81.52 | | 749 | 420 | 35.93 | 35.46 | 31.99 | 39.08 | 0.509 |  |  |  |  |
| Alcohol habits | Drunk up to 4 times/month | 209 | | 14.57 | | 147 | 62 | 29.67 | 31.24 | 23.44 | 40.28 |  |  |  |  |  |
|  | Drunk 2-6 times /week | 42 | | 2.93 | | 24 | 18 | 42.86 | 42.86 | 26.09 | 61.45 |  |  |  |  |  |
|  | Drunk every day | 14 | | 0.98 | | 10 | 4 | 28.57 | 27.01 | 9.39 | 56.13 |  |  |  |  |  |
| Smoking habits, smoking daily | No smoking at all | 1283 | | 89.35 | | 844 | 439 | 34.22 | 33.65 | 30.36 | 37.11 | 0.117 | ref |  |  |  |
|  | Smoking | 50 | | 3.48 | | 27 | 23 | 46.00 | 48.91 | 32.27 | 65.8 |  | 1.45 | 1.00 | 2.11 | 0.048 |
|  | Smoking every day | 103 | | 7.17 | | 61 | 42 | 40.78 | 40.83 | 29.83 | 52.84 |  | 1.21 | 0.90 | 1.64 | 0.212 |
|  |  |  | |  | |  |  |  |  | | |  |  |  |  |  |
|  |  | **Total** | |  | | **N CHIK  negative** | **N CHIK  positive** | **N CHIK  positive (%)** | **CHIK + weighed mean** | **95 % CI** | |  | **wPR** | **95 % CI** | | **P value** |
| Weight | (kg) | 1431 | |  | | 927 | 504 | 35.22 | 68.5 | 66.80 | 70.2 | 0.146 |  |  |  |  |
| Height | (meters) | 1420 | |  | | 922 | 498 | 35.07 | 1.61 | 1.60 | 1.62 | 0.650 |  |  |  |  |
| BMI (derived) | (Kg/m^2^) | 1415 | |  | | 918 | 497 | 35.12 | 26.6 | 25.90 | 27.30 | 0.289 |  |  |  |  |
