# Supplementary material for: Long lasting anti-IgG chikungunya seropositivity in the Mayotte population will not be enough to prevent future outbreaks: A seroprevalence study, 2019
Source: PLoS One. 2023 May 18;18(5):e0285879. doi: 10.1371/journal.pone.0285879 (PMC10194921; doi:10.1371/journal.pone.0285879)
Supplement: S6 File — (DOCX) [file pone.0285879.s006.docx]

**S6** Information on datasets, weights and variables used in the analysis

**DATASETS – Use of different files and weights to be applied, and variables list**

**Note:**

Considering the complexity of the weights applied in this study, we have opted to share three datasets to obtain the results reported in our manuscript. Each dataset has a different number of observations based on whether we report only the seroprevalence for the whole population (N= 2853), the seroprevalence by sex, age and sector (N=2778), or seroprevalence for all categories (age, sex, sector, origin, household, etc., N=1438) that have been used in our analysis. Before using each dataset, a STATA command that contains the weights (provided below for each dataset) and to be applied to obtain the presented results.

**1^st^ Dataset name:** **CHIK analysis_PLOSOne dataset_n2853**

N= 2853

Apply this STATA code/weight before any analysis:

**svyset IPSOS_NUM [pweight= poids_INF_VF], strata(IPSOS_STRATE) vce(linearized) singleunit(certainty)**

After running this command, it is possible to calculate **adjusted and weighed** **CHIK seroprevalence in the whole population**

**2^nd^ Dataset name:** **CHIK analysis_PLOSOne dataset_n2778**

N=2778

Apply this STATA code/weight before any analysis:

**svyset IPSOS_NUM [pweight= poids_QINF_VF], strata(IPSOS_STRATE) vce(linearized) singleunit(certainty)**

After running this command, it is possible to calculate **adjusted and weighed CHIK seroprevalence by sex, age and sector**, and run the univariate analysis for these variables with this number of observations.

**List of variables as listed in Table 1**

p15_cl_age2

P15_SEX

Sector

**3^rd^ Dataset name:** **CHIK analysis_PLOSOne dataset_n1438**

N=1438

Apply this STATA code/weight before any analysis:

**svyset IPSOS_NUM [pweight= poids_QLINF_VF], strata(IPSOS_STRATE) vce(linearized) singleunit(certainty)**

After running this command, it is possible to calculate **adjusted and weighed CHIK seroprevalence** considering all variables in the dataset.

**NOTE: THIS DATASET WAS USED FOR THE UNIVARIABLE AND MULTIVARIABLE ANALYSIS – SEE BELOW:**

- **LIST OF VARIABLES USED FOR THE UNIVARIABLE ANALYSIS,**
- **LIST OF VARIABLES USED FOR TESTING MULTICOLLINERARITY AND**
- **LIST OF VARIABLES USED FOR MULTIVARIABLE ANALYSIS**

**Based on statistical significance of the Pearsons’ Chi-square test (P-value ≤0.20), the following variables were selected for univariable analysis** (listed in the same order as in Table S2):

p15_cl_age2

P15_SEX

Sector

*(Note: age and sex showed a Pearson’s Chi2 with p higher than 0.2; however, it is common practice to include age and sex in every model to adjust always for these two factors; therefore, they have been included in all the models discussed in the manuscript)*

**/* sociodemographic characteristics*/**

p15_Socio12_GO

p15_enf_GO

p15_dipl_GO

p15_Socio12_GO

SOINS4_SOINS5

p15_ORIG_GO2

ORIG2_TimeInMayot

p15_nb_hab_GO

**/* household and environment*/**

p15_LOG1_hab

WatAcc3

WC_Latrine

WC_Latr2

Rivier_SeLaver

Riv_VaisLess

Dechets_Ext_Ferme

Dechets_Ext_Collectif

**/*Attitude to prevention for arboviral diseases*/**

GeneParMust

TransmPaluMust

PeurMalArbo

PaluMaladie

**/*Protective measures */**

ProtLotion

ProtDiffus

ProtVetem

ProtNet

ProtClimat

ProtRien

ProlElimPasConc

ProlifCouver

ProlifTrait

ProlifAucune

ProlifAutre

**/* health status*/**

S1_sante_percue

S3_Lim6mois

S2_MaladChro

p15_DIAB1_GO

p15_HTA1_GO

ALC3_4Cat

TAB1_TAB10

BMI_2Cat

PoidKg_INF

tailleMetre_INF

BMI_INF

**Based on p valued obtained via the abovementioned univariable analysis (P-value ≤0.20), the following variables were tested for multicollinearity. Below the list:**

p15_cl_age2

P15_SEX

Sector

p15_enf_GO

p15_dipl_GO

p15_Socio12_GO

SOINS4_SOINS5

p15_ORIG_GO2

ORIG2_TimeInMayot

p15_nb_hab_GO

p15_LOG1_hab

WC_Latrine

WaterAccess

Rivier_SeLaver

Riviere_Lessive

Riviere_Vaisselle

GeneParMust

TransmPaluMust

ProtLotion

ProtClimat

ProlifPasConc

ProlifCover

ProlifElim

S3_Lim6mois

PoidKg_INF

TAB1_TAB10

**Based on Spearman test for multicollinearity, the following variables were selected for running the models in the multivariable analysis (listed as in Table 2):**

p15_cl_age2

P15_SEX

Sector

p15_enf_GO

p15_dipl_GO

p15_Socio12_GO

SOINS4_SOINS5

p15_ORIG_GO2

p15_nb_hab_GO

p15_LOG1_hab

WC_Latr2

WatAcc3

Rivier_SeLaver

Riv_VaisLess

GeneParMust

TransmPaluMust

ProtLotion

ProtClimat

ProlElimPasConc

ProlifCouver

S3_Lim6mois

TAB1_TAB10
