# Supplementary material for: Long lasting anti-IgG chikungunya seropositivity in the Mayotte population will not be enough to prevent future outbreaks: A seroprevalence study, 2019
Source: PLoS One. 2023 May 18;18(5):e0285879. doi: 10.1371/journal.pone.0285879 (PMC10194921; doi:10.1371/journal.pone.0285879)
Supplement: Attachment — Submitted filename: Rebuttal letter _final_25April2023.docx [file pone.0285879.s008.docx]

***Manuscript: PONE-D-22-35076R1***

***Long lasting anti-IgG chikungunya seropositivity in the Mayotte population will not be enough to prevent future outbreaks: A seroprevalence study, 2019.***

**REBUTTAL LETTER**

**Reviewer #1**

*"The authors acknowledge your general feedback – thank you. We mentioned the evidence of not circulation of CHIKV in Mayotte in the discussion - please see lines 288-292."*

No report does not mean no circulation of the virus. Please add the point I have made in my previous comment for the discussion. The positive proportion remains the same compared to the previous study in 2006 means that there are no or minimal transmission of the virus since then. If there was an outbreak recently, the positive proportion must be way higher than the previous one. This is a stronger argument for the no circulation in the region point and should be added to the discussion.

**Answer:**

We entirely concur. Therefore, we made our agreement clearer by adding the following (lines 287-288): “This estimate is very similar to the seroprevalence of anti-CHIKV IgG as found in 2006 just after the outbreak in Mayotte*,* *suggesting that there was no or minimal transmission of the virus in the interim*.”

*"By using Spearman test we did consider multicollinearity rather than collinearity, as we checked the correlation by running the Spearman test with all the variables at once (although the output shows all possible combinations of correlation among each 2 variables). We did not opt for using the VIF command"*

No pair-wise co-linearity does not exclude the possibility of multicollinearity.
I do not get the difficulty to calculate VIF since the authors only need to calculate it based on the variables

**Answer:**

As the reviewer requested, we calculated the VIF with all the variables that we wanted to use for the multivariable analysis (see page 3 of S6), and we obtained an average VIF of 1.62, with a variable showing a maximum of 3.2 VIF. Based on this average VIF value we would have used all the variables for the models, but we felt that this value was not enough to highlight the collinearity issues that we felt very likely present among some of these variables. Therefore, we run the Spearman test, that actually showed us which couples of variables were showing collinearity. Based on this test, we then decided to combine those variables that appear to be collinear, and/or eliminate one of the two that showed collinearity. The final list of variables that we then used for the running our models is the one showed in page 4 of the S6 information sheet. We run again the VIF test with these and the final average value was 1.52, a bit lower than before as we did address the suspected collinearity issues.

We hope that this explanation clarifies why eventually we decided initially, to report the Spearman test on our manuscript rather than the VIF, which we felt, did not provide a satisfactory answer to whether there was collinearity and for which variables. We have added now a few sentences to reflect the request of the reviewer and this issue. We hope that this amendment is acceptable for the reviewer (see lines : 188-190).

*"Based on some reading on Spearman test, the use of it, and the interpretation of thresholds - see for instance these:*
[*https://geographyfieldwork.com/SpearmansRankCalculator.html*](https://geographyfieldwork.com/SpearmansRankCalculator.html)*,*
[*https://statistics.laerd.com/stata-tutorials/spearmans-correlation-using-stata.php*](https://statistics.laerd.com/stata-tutorials/spearmans-correlation-using-stata.php)
[*https://www.ncbi.nlm.nih.gov/pmc/articles/PMC3576830/*](https://www.ncbi.nlm.nih.gov/pmc/articles/PMC3576830/) *we opted for choosing 0.5 as the threshold. The range between 0.5 and 0.7 is generally associated to moderate correlation. As we did not want to have correlation as a factor that would somehow impact the analysis, 0.5 seemed to be an appropriate and conservative threshold to use (alongside a significant P value)."*

Please add these references to the manuscript to explain why you choose the number.

**Answer:**

As initially we understood from the reviewers that it was preferred not to use many web sites among the references, we opted to add only the manuscript among the above references (see No: 26)

*"We could not conduct an automated stepwise logistic regression using the stepwise command in STATA as the svy prefix command is not compatible with it. However, we did stepwise regression “manually”, specifically we used a backward stepwise process with the commands svy,:poisson and test."*

I am not familiar with STATA syntax so I cannot get what the authors trying to say here. Please use statistical terms to explain your reasoning.

**Answer:**

By "manual stepwise regression ", we meant that we followed the alternate procedure presented on the Stata website: William Sribney. 2022. Is there a way in Stata to do stepwise regression with svy: logit or any of the svy commands? Stata FAQ. StataCorp Available at: <https://www.stata.com/support/faqs/statistics/stepwise-regression-with-svy-commands/> Last Update Date: 16Nov2022. This website is referenced (see ref number: 27)

In brief, we arranged the selected covariates for each model into groupings ordered by decreasing presumed importance. Then we ran each full model before testing the last group of covariates. If not significant, the entire group was discarded. Otherwise, we kept the whole group. Then we repeated the procedure. Hopefully, adding it to our manuscript reference list will not pose a problem since we understood that the reviewers preferred that there were as few references to online resources as possible.

**Reviewer #2**:

Thank you for addressing the comments submitted by the reviewers. All of my comments and questions are now resolved.
